# The Impact of Axial CT Level Selection on Grading Trochlear Dysplasia Using Dejour Classification

**DOI:** 10.3390/diagnostics16010077

**Published:** 2025-12-25

**Authors:** Koray Kaya Kılıc, Mehmet Baris Ertan, Huseyin Selcuk, Tolga Kirtis, Oguzhan Uslu, Ozkan Kose

**Affiliations:** 1 Department of Radiology, Antalya Education and Research Hospital, Antalya 07010, Türkiye; koraykayakilic@yahoo.com; 2 Orthopedics and Traumatology Clinic, Private Medikum Hospital, Antalya 07350, Türkiye; 3 Department of Orthopedics and Traumatology, Van Education and Research Hospital, Van 65710, Türkiye; 4 Orthopedics and Traumatology Clinic, Tokat State Hospital, Tokat 60230, Türkiye; 5 Department of Orthopedics and Traumatology, Antalya Education and Research Hospital, Antalya 07010, Türkiye

**Keywords:** patellar dislocation, trochlear dysplasia, computed tomography, reliability, observer variation, knee joint

## Abstract

**Purpose:** The purpose of this study was to investigate how the choice of axial CT level affects the reliability and diagnostic accuracy of the Dejour classification for trochlear dysplasia and to evaluate a novel level defined at the most superior extent of the Blumensaat line. **Materials and methods:** Patients who presented with patellar instability or acute patellar dislocation between 2014 and 2024 and had preoperative CT scans were retrospectively reviewed. Fifty patients were randomly selected based on an a priori sample size calculation. For each knee, four axial CT levels were reconstructed: midpatellar level, Roman arc level, 3 cm above the joint line, and the top of the Blumensaat line. A consensus Dejour grade (A–D) was established by an experienced musculoskeletal radiologist and an orthopedic sports surgeon and used as the reference standard. Two orthopedic surgeons independently graded all 200 axial images twice at least 15 days apart. Quadratic weighted kappa (κ) with 95% confidence intervals (CI) was used to assess intra- and inter-observer reliability and agreement with the consensus. Diagnostic accuracy was defined as the proportion of correctly classified cases relative to the consensus and was compared across levels using Cochran’s Q test. **Results:** When all four levels were combined, intra-observer reliability was almost perfect for both observers (κ = 0.96 and 0.84; exact agreement 91% and 84%), and inter-observer reliability was substantial to almost perfect (κ = 0.72 and 0.78; exact agreement 72–73%). Agreement with the consensus across all levels was moderate (κ = 0.52–0.58; exact agreement 51–52%). Analyzing levels separately, intra-observer κ remained high at all levels, whereas inter-observer agreement and agreement with the consensus varied markedly. The midpatellar level showed only moderate inter-observer reliability and fair-to-moderate agreement with the consensus (κ = 0.36; accuracy 34–40%), whereas the top of the Blumensaat line showed the highest agreement with the consensus (κ 0.69) and the highest accuracy (up to 64%; pooled 61%); however, statistically significant between-level differences were detected in only one observer–time comparison. The 3 cm above the joint line and the Roman arc level demonstrated intermediate performance. **Conclusions:** Although intra-observer reliability of the Dejour classification is high regardless of axial CT level, both inter-observer agreement and diagnostic accuracy depend strongly on the selected slice. The axial CT level at the top of the Blumensaat line showed a consistent trend toward higher agreement and accuracy relative to the consensus standard and may be used as a standardized reference slice within routine multi-slice CT assessment to improve reproducibility; however, it should complement comprehensive imaging review and clinical evaluation.

## 1. Introduction

Patellofemoral dislocation is a relatively common knee injury, with an overall incidence of about 23–42 per 100,000 person-years in the general population. The risk peaks in adolescence, reaching approximately 148 per 100,000 person-years in 14–18-year-olds and 108 per 100,000 person-years in 10–17-year-olds. After a first dislocation, roughly 20–25% of patients experience recurrent episodes over 10 years, with recurrence rates of about one-third in the youngest age group [1,2,3]. Patellofemoral dislocation most often occurs in the presence of underlying anatomical and biomechanical abnormalities that predispose the patella to lateral instability. Typical anatomical risk factors include trochlear dysplasia, patella alta, increased tibial tubercle–trochlear groove (TT–TG) distance, excessive patellar tilt and lateralization, genu valgum with an increased Q-angle, increased femoral anteversion, tibial torsional abnormalities, and generalized ligamentous laxity with a hypermobile patella [4,5,6]. Therefore, a careful, systematic evaluation of all these risk factors is essential when planning treatment for patellofemoral dislocation [4,7,8].

Trochlear dysplasia is widely regarded as one of the most critical predisposing factors for patellofemoral dislocation [9,10]. A shallow or flattened trochlear groove provides insufficient bony constraint to maintain patellar tracking during knee flexion, thereby facilitating lateral translation and dislocation of the patella, particularly under valgus and rotational loads. The severity of trochlear dysplasia correlates with both the likelihood of first-time dislocation and the risk of recurrent instability, and it often coexists with other abnormalities such as patella alta and increased TT–TG distance [10,11]. Consequently, accurate assessment of trochlear morphology using standardized radiographic and cross-sectional imaging is a key component of risk stratification. It plays a central role in surgical decision-making, including the indication for trochleoplasty in selected high-risk patients [7,8,12].

The classification of trochlear dysplasia was first described on true lateral conventional radiographs and later refined with the advent of axial MRI and CT, which allow a more detailed assessment of trochlear morphology. Early work by Maldague and Malghem established a radiographic framework based solely on the appearance of the femoral trochlea on lateral views [13,14]. This concept was subsequently expanded to incorporate the supratrochlear region and to propose a classification that ultimately evolved into the widely used four-grade Dejour system [15,16,17,18]. This system not only standardizes the description of trochlear morphology but also plays a key role in guiding the management of patients with patellofemoral instability [7,12,19].

However, both lateral radiograph–based and axial imaging–based evaluations have important methodological limitations. Obtaining and interpreting a perfectly true lateral radiograph is technically demanding and associated with relatively low interobserver reliability, prompting a shift toward axial imaging [20,21,22,23]. Yet axial assessment of trochlear dysplasia has also lacked standardization, particularly regarding the axial level at which measurements are performed. Traditionally, the chosen slice depended on patellar height or the presence of the so-called “Roman arch” in the intercondylar notch [16,24], while Pfirrmann et al. [25] suggested evaluating the trochlea at a fixed level, 3 cm above the joint line. A systematic review by Dejour and co-workers showed that, among 11 included studies, 4 did not even report the axial level used, and 5 different slice levels were reported in the remaining 8 studies [21]. More recently, Tscholl and colleagues [20] demonstrated that varying the axial level within the same knee can yield different Dejour grades, underscoring the sensitivity of trochlear dysplasia classification to the choice of imaging level and highlighting the need for standardized assessment protocols.

Our primary hypothesis was that defining a standardized CT axial level at the most superior extent of the Blumensaat line would improve the diagnostic accuracy of the Dejour classification for trochlear dysplasia, compared with previously used axial levels. Accordingly, the aim of this study was to compare interobserver reliability and diagnostic accuracy of the Dejour classification across different CT axial levels and to determine the effect of this novel Blumensaat-based CT level on the assessment of trochlear dysplasia.

## 2. Materials and Methods

### 2.1. Patients and Study Design

A retrospective review was conducted of patients who presented to our institution with recurrent patellar instability or acute patellar dislocation between 2014 and 2024. Patients with a documented history of prior knee surgery prior to admission and/or without an available preoperative CT scan were excluded from the study. From the remaining eligible cohort, 50 patients with preoperative CT were randomly selected based on the a priori sample size calculation described below. All CT scans were obtained as part of preoperative evaluations during routine clinical care; no postoperative CT scans were acquired for this study. Direct radiographs and CT of these patients were evaluated and classified by a radiologist with at least 10 years of experience in musculoskeletal imaging and an orthopedic surgeon with at least 10 years of experience in sports trauma surgery, with a consensus decision. For the consensus reference classification, the musculoskeletal radiologist and the orthopedic surgeon reviewed the complete CT dataset using multiplanar reconstructions and considered all four predefined axial levels (Levels 1–4) together before assigning a single Dejour grade; no single preferred axial level was preselected for the reference standard. This consensus grade, therefore, represents a pragmatic, consensus-based reference rather than an independent gold standard. The study was conducted in accordance with the ethical principles of the Declaration of Helsinki, and approval was obtained from the hospital’s ethics committee (Approval Date and Issue: 22 February 2024/15-2).

### 2.2. Sample Size Calculation

The required sample size was estimated using the weighted kappa statistic as the primary outcome, based on the agreement between each observer and the reference Dejour classification. A previous study reported a mean intra-observer kappa of 0.52 for the Dejour classification on CT, which was taken as the null value [26]. For the present study, we assumed that almost perfect agreement, corresponding to a kappa of 0.80, would be clinically relevant [27]. The distribution of the four Dejour categories (A–D) was assumed to be approximately equal. Using these assumptions and a two-sided significance level of 0.05 with 80% statistical power, the minimum required sample size was calculated to be 41 patients. To account for possible exclusions and missing data, we planned to include 50 patients in the study.

### 2.3. Imaging and Determination of CT Levels

**Figure 1:**
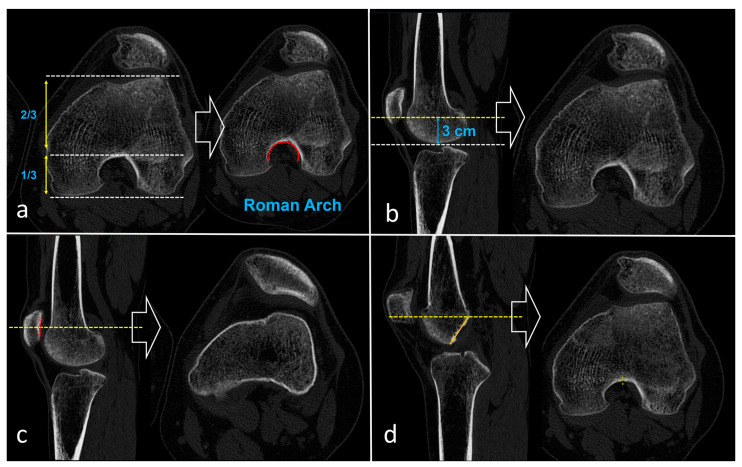
Determination of the four axial CT levels used for Dejour classification. (**a**) Roman arc level (Level 2): on the sagittal image, the distal femur is divided into a lower one-third and upper two-thirds, and the axial slice is chosen where the intercondylar notch resembles a Roman arch (red arc). (**b**) “3 cm above the joint line” level (Level 3): on the sagittal plane, a line is drawn 3 cm above the joint line (blue bracket), and the corresponding axial slice is obtained. (**c**) Midpatellar level (Level 1): the axial image is taken at the level passing through the midpoint of the patella on the sagittal reconstruction. (**d**) Top of the Blumensaat line level (Level 4): the axial slice is selected at the level tangent to the most superior point of the roof of the intercondylar notch (yellow line), corresponding approximately to the MPFL femoral footprint and the superior trochlea.

All computed tomography (CT) examinations were conducted with the same CT device (Siemens go. Up, Siemens, Munich, Germany) installed in the radiology department. The images were subsequently analyzed in DICOM format using the software program with a workstation (Sectra Workstation IDS7, Sectra AB, Linköping, Sweden). Given the variation in knee flexion angles across the CT images, a multiplanar reconstruction (MPR) was initially performed to obtain true axial images. These axial images were then reconstructed perpendicular to the femur’s anatomic axis in both the sagittal and coronal planes. Axial images were obtained at four distinct levels. The first axial section passes through the midpoint of the patella in the sagittal plane (Level 1). The second axial section was determined using the Roman arc method (Level 2). The third section is the axial image obtained 3 cm above the joint line (Level 3) [16,24,25]. Lastly, a cross-section was obtained that was tangent to the highest point of the ceiling of the intercondylar notch (Level 4) (Figure 1). The ‘top of the Blumensaat line’ level (Level 4) was identified on sagittal MPR as the axial slice tangent to the most superior point of the intercondylar notch roof. Because knee flexion varied across examinations, MPR was used to reconstruct true axial images perpendicular to the femoral anatomic axis, thereby standardizing level selection. Slice selection for the predefined levels, including Level 4, was performed in consensus by the same two reference readers. These images were then saved for the reliability study. This final section is the one proposed by the authors, which was hypothesized as the optimal evaluation of trochlear dysplasia. This section passes just below the distal femoral epiphysis and approximately at the level of the MPFL femoral footprint.

### 2.4. Inter and Intra-Observer Reliability and Validity Assessments

Two orthopedic surgeons, who are particularly interested in sports traumatology and have at least five years of clinical experience, participated in the study. The observers accessed the 200 images (50 patients × 4 levels per patient) digitally. A single axial image was provided to the observers. For the purpose of this reliability experiment, observers were intentionally restricted to a single representative axial slice per predefined level; this does not imply that a single axial slice is sufficient for clinical diagnosis or decision-making. In contrast, the consensus reference readers were not restricted to a single axial slice; they used the full CT series (with MPR) and evaluated the four predefined levels in combination to determine the consensus Dejour grade. Before the commencement of the study, a comprehensive briefing on the Dejour classification was provided, and the observers were permitted to review the figures and explanations pertaining to the classification during the observation period. Each observer assessed the images twice at 15-day intervals. Observers were blinded to their own and the other observer’s assessments to ensure impartiality. To maintain a randomized order, 200 images were shuffled on each occasion. Additionally, metadata, such as patient names and dates, were deleted from the images to prevent any potential recall bias.

### 2.5. Statistical Analysis

Statistical analyses were performed using IBM SPSS Statistics for Windows, Version 23.0 (IBM Corp., Armonk, NY, USA). Categorical variables were summarized as frequencies and percentages, and continuous variables as means with standard deviations. The Dejour classification was treated as an ordinal variable (grades A–D). Inter- and intra-observer reliability of the Dejour grades was assessed using quadratic weighted kappa (κ) coefficients with 95% confidence intervals (CI). For each of the four axial CT levels and for all levels combined, intra-observer reliability was evaluated by comparing the two readings of each observer, inter-observer reliability by comparing the two observers at each reading, and agreement with the reference standard by comparing each observer’s ratings at both time points with the consensus classification. Interpretation of κ values was performed according to the criteria proposed by Landis and Koch, with agreement classified as slight (κ = 0.00–0.20), fair (κ = 0.21–0.40), moderate (κ = 0.41–0.60), substantial (κ = 0.61–0.80), and almost perfect (κ = 0.81–1.00) [27].

Diagnostic accuracy was defined as the proportion of correctly classified cases relative to the consensus Dejour grade. For each observer and each reading, accuracy was calculated at each axial level together with 95% CIs derived from bootstrap resampling. Differences in accuracy among the four axial levels were examined separately for each observer and time point using Cochran’s Q test for related proportions. In addition, pooled accuracy across all observer–time combinations was calculated for each level and reported descriptively without formal hypothesis testing. This approach was preferred to limit multiplicity-related overinterpretation and to avoid inferential testing on pooled observations that are not fully independent across repeated readings. A two-sided *p*-value < 0.05 was considered statistically significant.

## 3. Results

### 3.1. Demographic Characteristics of Participants

A total of 50 patients were included in the study. The mean age of the cohort was 19.2 ± 5.8 years (range, 10–42 years). Twenty-three patients (46%) were male and 27 (54%) were female. The right knee was affected in 20 cases (40%), whereas the left knee was affected in 30 cases (60%).

### 3.2. Reliability of the Dejour Classification

Across all four axial CT levels combined, intra-observer reliability was almost perfect for both observers (Observer A: κ = 0.957, 95% CI 0.933–0.980; Observer B: κ = 0.838, 95% CI 0.767–0.909), with exact agreement rates of 91% and 84%, respectively. Inter-observer reliability was substantial at both reading sessions (T1: κ = 0.717, 95% CI 0.623–0.811; T2: κ = 0.784, 95% CI 0.706–0.860), with exact agreement of 72–73%. Agreement between each observer and the consensus ratings was moderate across both time points (κ ranging from 0.518 to 0.582, exact agreement 51–52%) (Table 1).

When the four axial CT levels were analyzed separately, intra-observer reliability remained high at all levels. Mean intra-observer κ (averaged over both observers) ranged from 0.840 (95% CI 0.704–0.927) at the midpatellar level to 0.921 (95% CI 0.841–0.980) at the 3 cm above the joint line level. Interobserver reliability (averaged over the two readings) was highest at the Roman arc level (κ = 0.798, 95% CI 0.630–0.935) and the Blumensaat line level (κ = 0.740, 95% CI 0.577–0.875), whereas the midpatellar level showed only moderate interobserver agreement (κ = 0.559, 95% CI 0.302–0.731). Agreement with the consensus classification varied substantially between levels. The highest mean κ versus the consensus was observed at the Blumensaat line level (κ = 0.692, 95% CI 0.525–0.811), followed by the 3 cm above the joint line level (κ = 0.591, 95% CI 0.373–0.775) and the Roman arc level (κ = 0.568, 95% CI 0.392–0.715). The midpatellar level showed only fair agreement with the consensus (κ = 0.363; 95% CI: 0.202–0.529). Thus, while intra-observer agreement was high at all levels, both interobserver reliability and agreement with the reference standard were clearly lowest at the midpatellar level and highest at the Blumensaat line level (Table 2).

### 3.3. Diagnostic Accuracy According to Axial CT Level

Diagnostic accuracy is defined as the proportion of cases correctly classified relative to the consensus classification. Diagnostic accuracy of the Dejour classification relative to the consensus standard is summarized in Table 3. For Observer A at the first reading, a statistically significant difference in accuracy among the four axial levels was detected (Cochran’s Q *p* = 0.010), with the highest proportion of correctly classified cases at the top of the Blumensaat line and the lowest at the midpatellar level. Although subsequent readings for both observers did not show statistically significant differences between levels, a consistent pattern was observed across all reader–time combinations, with the top of Blumensaat’s line and the 3 cm above the joint line level yielding higher accuracy, while the midpatellar level remained the least accurate. When all readings were pooled, overall accuracy was again lowest at the midpatellar level and highest at the top of the Blumensaat line level. These pooled estimates are presented descriptively and should not be interpreted as proof of statistical superiority across levels.

## 4. Discussion

The present study demonstrates that, while the Dejour classification of trochlear dysplasia shows almost perfect intra-observer reliability across axial CT levels, inter-observer agreement and diagnostic accuracy are highly dependent on slice selection. When all levels were pooled, inter-observer reliability appeared substantial to almost perfect; however, level-specific analyses revealed marked variability, with the midpatellar level performing worst (lowest κ values, poorest agreement with the consensus classification, and lowest accuracy). In contrast, the axial section at the top of the Blumensaat line showed a consistent directional trend toward higher agreement with the reference standard and a higher proportion of correctly classified cases; however, statistically significant between-level differences in diagnostic accuracy were observed in only one observer–time comparison. Collectively, these findings indicate that the apparent robustness of the Dejour system within individual readers may conceal clinically relevant susceptibility to level selection, and that lack of level standardization may contribute to misclassification, heterogeneous reporting, and variability in surgical indications. Although the proposed Blumensaat-referenced level is intended to improve grading consistency, treatment decisions should remain based on comprehensive imaging review and clinical context, including other established anatomic risk factors. Finally, while this investigation was CT-based, the need for standardized slice selection may also be relevant to MRI-based assessments; nonetheless, modality-specific differences warrant dedicated validation on MRI.

**Figure 2:**
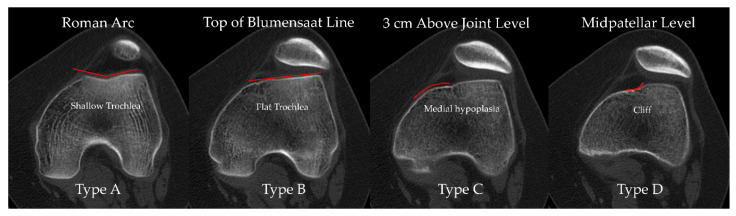
Axial CT images from the same patient demonstrate how Dejour trochlear dysplasia grading varies according to the axial level. From left to right: at the Roman arc level the trochlea appears shallow and would be classified as Type A; at the level of the top of the Blumensaat line it appears flat (Type B); at 3 cm above the joint line there is medial trochlear hypoplasia consistent with Type C; and at the midpatellar level a “cliff” pattern suggests Type D. Red lines outline the trochlear articular surface and highlight the level-dependent variation in morphology.

Previous CT-based studies have typically relied on three main axial levels for Dejour grading: the midpatellar level, a fixed level 3 cm above the joint line, and the so-called Roman arc level [16,24,25]. However, each of these has inherent limitations that may explain the suboptimal accuracy observed in our analysis. The midpatellar level is strongly influenced by patellar height; in patients with patella alta, this slice is shifted proximally and may even lie cranial to the trochlear cartilage, such that the true trochlear morphology is incompletely or not at all visualized, which likely accounts for its poorest diagnostic performance in our series (Figure 2). Similarly, the “3 cm above the joint line” level, although seemingly standardized, is in fact dependent on patient size: in large individuals, this fixed distance often corresponds to the deeper portion of the trochlear groove, whereas in smaller or adolescent patients it may intersect a more proximal segment of the trochlea, again introducing systematic variability in the anatomy being assessed. The Roman arc level is likewise subject to observer dependence, as the apparent configuration of the arc changes across consecutive slices, and determining the “1/3–2/3” relationship requires an additional measurement step. In contrast, the level we propose—defined at the most superior extent of the Blumensaat line—is straightforward to identify without ancillary measurements, can be consistently localized on both sagittal and coronal reconstructions, and is anatomically close to the femoral footprint of the MPFL and the superior trochlea, which represents the primary patellofemoral contact area in early flexion (0–30°) [28,29]. These features likely contribute to the superior combination of reliability and diagnostic accuracy observed at this level in the present study.

Several recent reports have highlighted that, although the Dejour classification remains the most widely used framework for describing trochlear dysplasia, its reliability is far from uniform and is strongly influenced by methodological factors. Saccomanno et al. reported in a systematic review that reliability coefficients for Dejour grading span a wide range, from poor to very good, across modalities and studies, with most series judged to be at high risk of bias owing to heterogeneous imaging protocols and suboptimal study design [23]. Kazley and Banerjee similarly concluded that the Dejour system suffers from inconsistent grading of severity, limited reproducibility, and questionable usefulness in guiding treatment decisions [30]. More specifically, Tscholl et al. [20] and, more recently, Pineda et al. [21] emphasized that observer agreement for Dejour types is highly sensitive to the imaging modality and, crucially, to the choice of axial slice, with studies using different levels or incompletely defined protocols reporting only slight to moderate inter-observer agreement in some settings. Tanaka’s editorial further underscored that trochlear dysplasia is “difficult to measure, no matter how you slice it,” and called for standardized slice selection as a prerequisite for more reliable quantification [31]. Within this context, our study was specifically designed to disentangle the effect of axial CT level from the intrinsic properties of the Dejour system by directly comparing reliability and diagnostic accuracy across commonly used levels and a novel Blumensaat-based level, using a consensus grade as reference. Our findings refine the current understanding of Dejour reliability by demonstrating that the classification itself can be highly consistent within observers but that its practical performance is critically dependent on standardized axial level selection—an issue our proposed Blumensaat-based level appears to mitigate. In parallel with our findings, Brumini et al. recently reported that, even in MRI-based quantitative assessment of patellar dislocation, some commonly used metrics (e.g., sulcus angle and trochlear depth) can be highly discriminative yet demonstrate lower inter-rater agreement, whereas lateral trochlear inclination (LTI) showed excellent reproducibility together with very high diagnostic performance. This highlights that measurement reliability remains sensitive to methodological choices (landmark definition and the imaging level/slice used), reinforcing the need for standardized assessment protocols across modalities [32].

A major strength of this study is its focused, methodologically robust design, specifically aimed at isolating the effect of axial CT level on the reliability and diagnostic accuracy of the Dejour classification. Four clearly defined and reproducible CT levels, including the novel level at the most superior extent of the Blumensaat line, were evaluated in the same cohort, enabling a direct within-subject comparison rarely available in the existing literature. Additional methodological strengths include the use of a priori sample size calculation based on weighted kappa as the primary outcome, standardized image acquisition on a single CT system with multiplanar reconstruction to obtain true axial slices, and a consensus reference classification established jointly by an experienced musculoskeletal radiologist and an orthopedic sports surgeon. Furthermore, reliability was comprehensively assessed using quadratic weighted kappa and 95% confidence intervals, with two independent orthopedic observers performing blinded, repeated readings, while diagnostic accuracy and differences between levels were formally tested.

This study also has limitations that should be acknowledged. First, it is a single-center study with a relatively modest sample size of 50 patients, although this number was statistically justified and adequate for the planned analyses. Second, CT was the sole imaging modality evaluated; given the increasing emphasis on MRI-based assessment and quantitative trochlear measurements, extrapolation of these results to MRI must be made with caution. Recent MRI-based reliability data further emphasize that reproducibility may vary substantially across measurements, supporting the rationale for modality-specific standardization and motivating future validation of a Blumensaat-referenced level on MRI-based Dejour grading, in combination with quantitative MRI metrics [32]. Third, the consensus Dejour grade was used as the reference standard, but it remains a subjective construct rather than an independent external gold standard, and the classification itself is qualitative and does not capture the full morphologic spectrum of trochlear dysplasia. Accordingly, the ‘diagnostic accuracy’ results in this study should be interpreted as agreement with a consensus-based reference classification, not as accuracy against an independent objective gold standard. Fourth, only two experienced observers with a particular interest in sports traumatology were included; reliability across a broader range of readers with varying levels of experience was not assessed. Finally, we did not separately quantify the inter- or intraobserver reproducibility of identifying the ‘top of the Blumensaat line’ level itself. Although MPR-based reconstruction was used to standardize true axial alignment, the ease and reproducibility of selecting this landmark may differ across CT acquisition/reconstruction protocols, knee flexion positions, and in younger/skeletally immature knees. Therefore, external validation of landmark identification reproducibility across different protocols and age groups is warranted

## 5. Conclusions

The present study shows that while the Dejour classification of trochlear dysplasia exhibits almost perfect intra-observer reliability across axial CT levels, its inter-observer agreement and diagnostic accuracy are highly dependent on the selected slice. Among the evaluated levels, the axial section at the most superior extent of the Blumensaat line provided a favorable balance of inter-observer reliability and agreement with a consensus reference. It showed a consistent trend toward higher diagnostic accuracy. Nevertheless, statistically significant between-level accuracy differences were detected in only one observer–time comparison; therefore, these findings should be interpreted as supportive but not definitive evidence of superiority. These findings support the use of the Blumensaat-based level as a standardized reference slice for CT-based Dejour grading in both clinical practice and research. This proposed reference level is intended to standardize slice selection for Dejour grading and does not replace a comprehensive evaluation of the whole CT series and the overall clinical context. Future investigations should validate this level in larger, multicenter cohorts and across different imaging modalities, particularly MRI, and explore its integration with emerging quantitative trochlear metrics and three-dimensional assessment techniques.

## Figures and Tables

**Table 1:** Overall reliability of assessments.

Comparisons	κ (95% CI)	Exact Agreement	Interpretation
Intra-observer A (T1 vs. T2)	0.957 (95% CI 0.933–0.980)	91%	Almost perfect
Intra-observer B (T1 vs. T2)	0.838 (95% CI 0.767–0.909)	84%	Almost perfect
Inter-observer (T1: A vs. B)	0.717 (95% CI 0.623–0.811)	72%	Substantial
Inter-observer (T2: A vs. B)	0.784 (95% CI 0.706–0.860)	73%	Substantial
Observer A vs. Consensus (T1)	0.580 (95% CI 0.481–0.679)	52%	Moderate
Observer A vs. Consensus (T2)	0.574 (95% CI 0.473–0.674)	52%	Moderate
Observer B vs. Consensus (T1)	0.518 (95% CI 0.407–0.628)	51%	Moderate
Observer B vs. Consensus (T2)	0.534 (95% CI 0.424–0.643)	52%	Moderate

Abbreviations: T1: First Time, T2: Second Time, A: Observer A, B: Observer B, CI: Confidence Interval.

**Table 2:** Reliability of each CT level.

CT Level	Comparisons	κ (95% CI)	Exact Agreement	Interpretation
Roman Arc	Intra-observer A (T1 vs. T2)	0.958 (95% CI 0.897–0.991)	92%	Almost perfect
	Intra-observer B (T1 vs. T2)	0.788 (95% CI 0.542–0.977)	90%	Substantial
	Inter-observer (T1: A vs. B)	0.760 (95% CI 0.545–0.934)	84%	Substantial
	Inter-observer (T2: A vs. B)	0.836 (95% CI 0.700–0.948)	84%	Almost perfect
	Observer A vs. Consensus (T1)	0.565 (95% CI 0.389–0.736)	54%	Moderate
	Observer A vs. Consensus (T2)	0.572 (95% CI 0.385–0.735)	54%	Moderate
	Observer B vs. Consensus (T1)	0.561 (95% CI 0.353–0.751)	52%	Moderate
	Observer B vs. Consensus (T2)	0.574 (95% CI 0.381–0.743)	52%	Moderate
3 cm Above Joint Line	Intra-observer A (T1 vs. T2)	0.969 (95% CI 0.928–0.993)	92%	Almost perfect
	Intra-observer B (T1 vs. T2)	0.874 (95% CI 0.718–0.977)	92%	Almost perfect
	Inter-observer (T1: A vs. B)	0.688 (95% CI 0.486–0.863)	70%	Substantial
	Inter-observer (T2: A vs. B)	0.779 (95% CI 0.588–0.925)	72%	Substantial
	Observer A vs. Consensus (T1)	0.603 (95% CI 0.359–0.783)	56%	Substantial
	Observer A vs. Consensus (T2)	0.627 (95% CI 0.392–0.809)	54%	Substantial
	Observer B vs. Consensus (T1)	0.533 (95% CI 0.276–0.738)	54%	Moderate
	Observer B vs. Consensus (T2)	0.602 (95% CI 0.368–0.802)	58%	Substantial
Midpatellar Level	Intra-observer A (T1 vs. T2)	0.921 (95% CI 0.848–0.974)	86%	Almost perfect
	Intra-observer B (T1 vs. T2)	0.759 (95% CI 0.479–0.925)	78%	Substantial
	Inter-observer (T1: A vs. B)	0.475 (95% CI 0.197–0.727)	54%	Moderate
	Inter-observer (T2: A vs. B)	0.644 (95% CI 0.399–0.825)	62%	Substantial
	Observer A vs. Consensus (T1)	0.421 (95% CI 0.222–0.606)	34%	Moderate
	Observer A vs. Consensus (T2)	0.394 (95% CI 0.190–0.576)	38%	Fair
	Observer B vs. Consensus (T1)	0.322 (95% CI 0.139–0.496)	40%	Fair
	Observer B vs. Consensus (T2)	0.315 (95% CI 0.130–0.488)	40%	Fair
Top of Blumensaat Line	Intra-observer A (T1 vs. T2)	0.952 (95% CI 0.856–1.000)	94%	Almost perfect
	Intra-observer B (T1 vs. T2)	0.766 (95% CI 0.600–0.894)	76%	Substantial
	Inter-observer (T1: A vs. B)	0.760 (95% CI 0.561–0.919)	80%	Substantial
	Inter-observer (T2: A vs. B)	0.719 (95% CI 0.520–0.880)	74%	Substantial
	Observer A vs. Consensus (T1)	0.727 (95% CI 0.567–0.848)	64%	Substantial
	Observer A vs. Consensus (T2)	0.692 (95% CI 0.486–0.840)	62%	Substantial
	Observer B vs. Consensus (T1)	0.674 (95% CI 0.471–0.823)	58%	Substantial
	Observer B vs. Consensus (T2)	0.674 (95% CI 0.472–0.822)	58%	Substantial

Abbreviations: T1: First Time, T2: Second Time, A: Observer A, B: Observer B, CI: Confidence Interval.

**Table 3:** Accuracy of the Dejour classification according to axial CT level.

Comparison	Roman Arc (%, 95% CI)	3 cm Above the Joint Line (%, 95% CI)	Midpatellar Level (%, 95% CI)	Top of Blumensaat Line (%, 95% CI)	*p*-Value *
Observer A, T1	54% (40–68)	56% (42–70)	34% (20–48)	64% (50–78)	0.010
Observer A, T2	54% (40–68)	54% (40–68)	38% (24–52)	62% (48–76)	0.075
Observer B, T1	52% (38–66)	54% (40–68)	40% (26–54)	58% (44–70)	0.277
Observer B, T2	52% (38–66)	58% (44–72)	40% (26–54)	58% (44–70)	0.190
Pooled #	53% (40–66)	56% (44–68)	38% (7–50)	61% (49–72)	—

Abbreviations: T1: First Time, T2: Second Time, A: Observer A, B: Observer B, CI: Confidence Interval; * Cochran’s Q test, # All readers and readings.

## Data Availability

The datasets presented in this article are not readily available because of privacy, ethical, and legal restrictions protecting patient confidentiality. Requests to access the datasets should be directed to corresponding author.

## Abbreviations

CI	Confidence interval
CT	Computed tomography
DICOM	Digital Imaging and Communications in Medicine
MPFL	Medial patellofemoral ligament
MRI	Magnetic resonance imaging
MPR	Multiplanar reconstruction
SD	Standard deviation
SPSS	Statistical Package for the Social Sciences
T1	First reading/first time point
T2	Second reading/second time point
TT–TG	Tibial tubercle–trochlear groove
